# Differing environmental risk factors for membranous versus proliferative lupus nephritis

**DOI:** 10.1186/ar3957

**Published:** 2012-09-27

**Authors:** MA Dooley, CG Parks, GS Cooper

**Affiliations:** 1 University of North Carolina at Chapel Hill, NC, USA; 2 National Institutes of Environmental and Health Sciences, City Research Triangle Park, North Carolina, USA

## Objective

Nephritis is a frequent and severe organ involvement in patients with systemic lupus erythematosus. This study examined hormonal and environmental risk factors for biopsy-proven lupus nephritis (LN), comparing exposures of lupus patients with LN with patients without LN; additional analyses were conducted specifically for the subsets of those with membranous versus proliferative nephritis.

## Methods

The Carolina Lupus Study (CLU) is a population-based, case-control study of hormonal, environmental, and genetic risk factors for SLE in the southeastern US (*n *= 265 incident cases). Diagnosis of LN was determined at the time of enrollment up to 2001 by ACR criteria, with 63 nephritis cases confirmed based on renal biopsy records. An additional 100 biopsy-confirmed LN patients from the CLU study area and time period were identified through disease registries, comprising the nephritis CLU (CLN) cohort. Data collection for both cohorts was a structured 60-minute interview including female reproductive history, lifetime job history, smoking history, use of moonshine and family history of kidney disease, dialysis, hypertension and diabetes. We estimated the association between exposures and risk of developing LN, and LN subtypes (proliferative vs. membranous LN) using logistic regression models, with OR and 95% CI adjusted for age, state, race and education. Analyses were limited to exposures before diagnosis of lupus or LN.

## Results

Ninety-seven of the LN cases were classified as proliferative, 39 as membranous and 20 as other classifications (WHO class 2, 6, or unclear from medical record). In 56 patients (36%), diagnosis of LN was concurrent with diagnosis of SLE, in 53 patients (36%) within 2 years of diagnosis and in 47 patients (28%) >2 years after SLE diagnosis. Thirty percent of AA patients versus 10% Caucasians met ACR criteria for LN in the first year following diagnosis. Compared with lupus patients who did not develop nephritis, risk of LN was associated with older age at menarche (*P *for trend = 0.045), but no other reproductive factors. Reported use of moonshine was significantly higher among LN patients (OR = 3.3, 95% CI = 1.3 to 8.6), and higher among membranous compared with proliferative nephritis (OR = 17.87, 95% CI = 1.55 to 205.89). Likely or possible solvent exposure was associated with a significantly lower risk of nephritis (OR = 0.22, 95% CI = 0.06 to 0.76). There was no association with occupational exposure to silica dust, heavy metals or smoking history after adjusting for covariates. Null associations were seen for most other exposures and risk factors examined. See Table 1.

**Table 1:** Reproductive history among LN cases and lupus cases without nephritis stratified by current age (females only)

	Ages 18 to 34 (*n *= 127)		
		
Age at menarche	All LN cases (*n *= 64)	Lupus cases without nephritis (*n *= 63)	OR (95% CI)
8 to 9	5 (8)	3	1.84 (0.35 to 9.62)
10	3	7 (11)	0.54 (0.11 to 2.65)
11	5 (8)	8 (13)	0.58 (0.15 to 2.29)
12	11 (17)	18 (29)	0.76 (0.27 to 2.18)
13	18 (28)	19 (30)	1.00 (referent)
14	7 (11)	4	1.54 (0.34 to 6.93)
15	9 (14)	2	4.94 (0.88 to 27.92)
16	5 (8)	2	3.57 (0.56 to 22.73)
17 to 18	1	0	-
Trend test (*P *value)			(0.03)

Missing value (refused) for one lupus nephritis case; three lupus nephritis cases excluded because diagnosis occurred 3 to 4 years before menarche (at age 14); total *n *= 78.

## Conclusion

Our findings provide evidence of an increase risk of LN associated with past use of moonshine, family history of kidney disease, and later age at menarche. Null associations were seen for most other exposures and risk factors examined. Although not previously studied in relation to LN, moonshine use has been associated with chronic kidney disease in other studies as it contains lead and other heavy metals, and is thought to damage the kidney through a variety of mechanisms. The negative association of solvent exposure with membranous nephritis suggests that different pathophysiologic mechanisms may be involved in membranous versus proliferative LN.

